# A fast and sensitive isocratic high performance liquid chromatography method for determination of guaraná (*Paullinia cupana*) flavan-3-ols

**DOI:** 10.1016/j.mex.2019.04.008

**Published:** 2019-04-18

**Authors:** Lina Yonekura, Hirotoshi Tamura

**Affiliations:** Faculty of Agriculture, Kagawa University, 2393 Ikenobe, Miki-cho, Kagawa, 761-0795, Japan

**Keywords:** A fast and sensitive isocratic HPLC-UV method for determination of guaraná (*Paullinia cupana*) flavan-3-ols, Guaraná, *Paullinia cupana*, Catechin, Epicatechin, Procyanidin, Flavan-3-ol, HPLC-UV

## Abstract

Most methods for quantification of catechins in guaraná and other food matrices rely on lengthy gradients to resolve all peaks, and the analysis time for one sample is around 45–135 min. The present method is a fast, sensitive and simple HPLC-UV method, with a 16-minute isocratic run that deliver the high throughput needed to process a large number of samples without compromising the analyte stability. This method is suitable for the determination of catechins, procyanidins (up to degree of polymerisation 2) and caffeine in guaraná extracts, in vitro digesta, and Caco-2 cell permeates. The higher sensitivity was achieved by detection at 210 nm, after checking for the absence of interfering substances in the matrices. The method was validated and LODs of 0.019, 0.030, 0.028, 0.030, and 0.043 nmol/mL were achieved for catechin, epicatechin, procyanidin B1, procyanidin B2 and caffeine, respectively. Standard recovery at three different concentrations were within 97–109%. Intra- and inter-day variabilities (RSD) were both under 4%.

• This is an isocratic HPLC-UV method for the quantification of flavan-3-ols from guaraná (Paullinia cupana), which is 3–8 times faster and >80 times sensitive than previously published methods using UV or diode array detectors.
• It is cost-effective as it uses the widely available and affordable UV detector and consumes less solvent due the short analysis time.
• With low LODs, fast sample preparation and the short analytical run, this method can be used for the quantification of flavan-3-ols and caffeine in guaraná extracts as well as guaraná in vitro digesta and its Caco-2 permeates.

## Graphical abstract

**Figure:**
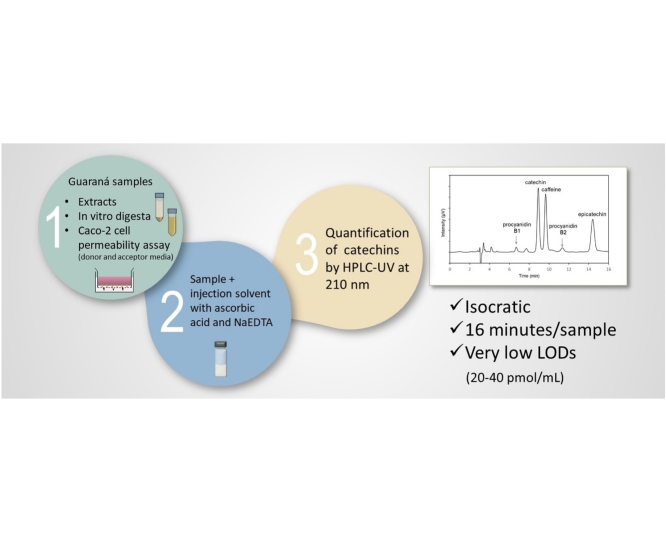

**Specifications Table**

**Table:** 

Subject Area:	Agricultural and Biological Sciences
More specific subject area:	Food analysis; determination of bioactives
Method name:	A fast and sensitive isocratic HPLC-UV method for determination of guaraná (Paullinia cupana) flavan-3-ols
Name and reference of original method:	Yonekura, L., Martins, C. A., Sampaio, G. R., Monteiro, M. P., César, L. A. M., Mioto, B. M., … Torres, E. A. F. da S. (2016). Bioavailability of catechins from guaraná (Paullinia cupana) and its effect on antioxidant enzymes and other oxidative stress markers in healthy human subjects. Food & Function, 7(7), 2970–2978. https://doi.org/10.1039/C6FO00513F Silva, C. P., Sampaio, G. R., Freitas, R. A. M. S., & Torres, E. A. F. S. (2018). Polyphenols from guaraná after in vitro digestion: Evaluation of bioacessibility and inhibition of activity of carbohydrate-hydrolyzing enzymes. Food Chemistry, 267, 405–409. https://doi.org/10.1016/j.foodchem.2017.08.078
Resource availability:	n.a.

## Method details

### Background

Flavan-3-ols are a class of flavonoids, which are very abundant in guaraná (Paullinia cupana) as well as other sources of such as cocoa, green tea and apple. Despite their high concentration in foods, their bioavailability is typically low, demanding sensitive methods for their quantification in samples from human bioavailability studies as well as Caco-2 permeability assays. High performance liquid chromatography (HPLC) coupled with high-sensitivity detectors such as coulometric, fluorescence or mass spectrometry are usually the choice for the determination of flavan-3-ols (also known as catechins) in bioavailability studies. However, these detectors are expensive and not usually available in smaller labs. The ultraviolet-visible (UV) detector is the most affordable and commonly available detector for HPLC setups, but the drawback of HPLC-UV methods is the low sensitivity of catechins at their typical and most selective absorbance maximum (280 nm). Catechin and epicatechin show absorbance maxima at 280 and 210 nm. The 280 nm band is associated with electron transitions of catechin’s A-ring benzoyl system, thus reasonably selective but with a low response. The 210 nm band is several times more responsive (i.e. giving high sensitivity) than the 280 nm band, however many organic compounds absorb at this wavelength, thus reducing the method’s selectivity.

Samples from Caco-2 cell permeation assays of guaraná in vitro digesta usually contain low catechin concentrations, but they are relatively ‘clean’ (i.e. without interfering peaks at low wavelengths).Therefore, detection at 210 nm would be an option to increase the method’s sensitivity. A sample of guaraná extract was previously analysed by HPLC with a photodiode array in the 190–450 nm range. The chromatogram showed that all peaks at 210 nm were from the same compounds at 280 nm (data not shown).

Our previous method [1], as well as most methods for quantification of catechins in guaraná [[2], [3], [4]] and other food matrices rely on lengthy gradients to resolve all peaks and to remove lipophilic compounds left behind in the column, and the analysis time for one sample ranges from 45 to 135 min. The present method is a fast, sensitive and simple HPLC-UV method, with a 16-minute isocratic run that deliver the high throughput needed to process a large number of samples without compromising the analyte stability. This method is suitable for the determination of catechins, procyanidins (up to degree of polymerisation 2) and caffeine in guaraná extracts, in vitro digesta, and Caco-2 cell permeates.

### Material and chemicals

Guaraná seeds were purchased at a local market in Manaus (Amazonas state, Brazil) and their geographical origin was Maués (Amazonas state). Seeds were pulverised with a blender and passed through a 250 μm sieve before extraction or in vitro digestion. (+)-Catechin and (-)-epicatechin were purchased from Nacalai Tesque (Japan), Procyanidin B1 from Cayman Chemical (USA), Procyanidin B2 from Extrasynthese (France) and caffeine from FUJIFILM-Wako (Japan). Acetonitrile was of HPLC grade and purchased from Sigma-Aldrich or Honeywell. All other chemicals were of reagent grade. Ultra-pure water (resistivity higher than 18 MΩ cm) was used in all experiments.

Injection solvent with preservatives (diluent for working standards and samples)

- 2 mM ascorbic acid, 0.05 mM NaEDTA and 0.1% formic acid in water.

Stock and working solutions of catechins, procyanidins and caffeine

- Purify a small amount of dimethyl formamide by passing it through a silica column just before the preparation of standards.
- Dissolve the required amount of compound in 0.4 M phosphate buffer (pH 3.6) containing 10% DMF, 20 mg/mL ascorbic acid and 1 mg/mL NaEDTA. Stock solutions of 2 μmol/mL are stable when stored at -20 °C for at least 6 months.
- Prepare working solutions for the calibration curves by serial dilution with the injection solvent. Calibration standards for catechin, epicatechin and caffeine had the following concentrations: 0.195, 0.391, 0.781, 1.563, 3.125, 6.25, 12.5, 25, 50 and 100 nmol/mL. The concentrations of calibration standards for procyanidin B1 and B2 were 0.098, 0.195, 0.391, 0.781, 1.563, 3.125, 6.25, 12.5, 25 and 50 nmol/mL.
- Inject 20 μL of each calibration standard into HPLC.
- Quantification was done by peak area, interpolated from 10-point calibration curves of (+)-catechin, (-)-epicatechin, procyanidin B1, procyanidin B2 and caffeine.

Guaraná seed powder extract

- Weigh one gram of guaraná powder into a 50 mL polypropylene screw capped tube.
- Add 20 mL methanol:water (30:70, v:v).
- Homogenise for 3 min with a Polytron® (Kinematica) probe homogeniser at 14,000 rpm, and centrifuge at 18,000×*g* for 15 min (centrifugation can be done at lower speeds).
- Collect the supernatant and repeat the extraction 3 additional times, as follows: 1 time with 20 mL methanol:water (30:70, v:v), and two times with 20 mL acetone:water (70:30, v:v).
- Combine all supernatants in a volumetric flask and adjust to 100 mL with water. Dilute 10–20-fold with the injection solvent.
- Inject 20 μL into HPLC.

Preparation of basal media samples for HPLC-UV analysis

- Pipette 10 μL of a 20× concentrated injection solvent (i.e. 40 mM ascorbic acid, 1 mM NaEDTA and 2% formic acid in water) into 200 μL of basal medium from Caco-2 permeation assays of guaraná digesta.
- Transfer into HPLC vials with inserts and inject 40 μL into HPLC.

Preparation of in vitro digesta samples for HPLC-UV analysis

- Dilute the guaraná in vitro digesta 10–20 fold with the injection solvent
- Inject 20 μL into HPLC for analysis

HPLC apparatus and analytical conditions

- Gulliver series (JASCO Corporation, Japan), composed of a DG-980 degasser, two PU-980 pumps, a HG-980-31 solvent mixing unit, an AS-950 auto-sampler, a CO-965 column oven, a UV-970 UV–vis detector, and a JASCO LC-NetII/ADC interface.
- Mobile phase: acetonitrile/water 12:88 (v:v), with 0.1% formic acid
- Column: Shiseido Capcell Pak column (UG 120 C18 250 × 4.6 mm, 5 μm) attached to a Waters Novapak C18 guard column.
- Mobile phase flow speed, 1 mL/min.
- Column oven temperature, 35 °C.
- Detection wavelength, 210 nm.
- Data acquisition time, 16 min.
- Perform quantification by interpolating the peak areas at 210 nm into the calibration curves.

### Method validation

Samples from a previous study [5] were used for method validation. Samples containing guaraná catechins were: guaraná extracts, in vitro digesta of guaraná seeds and basal media (Hanks’ balanced salt solution, HBSS) from Caco-2 permeability assays done with guaraná in vitro digesta.

**Fig. 1:**
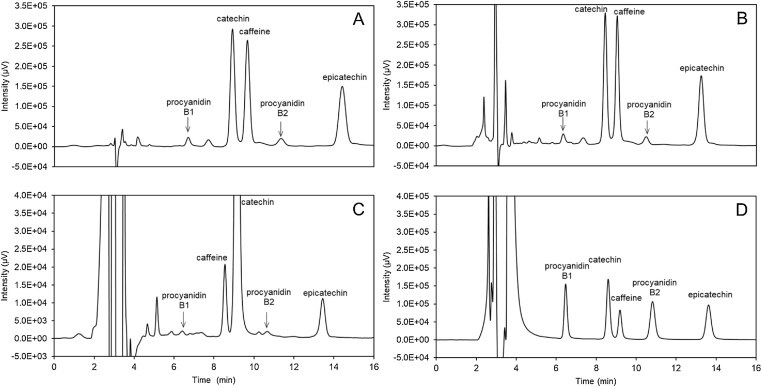
Typical HPLC-UV chromatograms of guaraná extract (A), in vitro digesta (B) and their Caco-2 cell permeates (C) and well as the standard mix (D), acquired at 210 nm.

Typical chromatograms from guaraná extracts, in vitro digesta and their Caco-2 cell permeates from bioaccessibility studies are shown on Fig. 1, and typical retention times are listed on Table 1.

**Table 1:** Retention time, linearity range, slope and correlation coefficient of calibration for catechins, procyanidins and caffeine by isocratic HPLC-UV at 210 nm.

Compound	Rt (min)	Linearity^a^ (nmol/mL)	Slope^b^ (×10^4^)	*r^2^*
Catechin	8.55	0.195 - 100	4.339	0.9997
Epicatechin	13.45	0.195 - 100	3.918	0.9993
Procyanidin B1	6.42	0.098 - 50	7.098	0.9997
Procyanidin B2	10.53	0.098 - 50	8.120	0.9993
Caffeine	9.16	0.195 - 100	2.161	0.9994

a Curve plotted from 20 μL injections of 10 standard solutions across the specified range.

b Slope of the regression equation *y = ax* where *y* is the peak area and *x* is the standard concentration in nmol/mL.

Method validation was performed as described by the International Conference on Harmonization (ICH) guidelines [6]. Linearity was confirmed throughout the 10-point calibration range of all five compounds, with correlation coefficients higher than 0.999 for all tested compounds as shown in Table 1. We chose to use a zero-intercept equation) as it did not cause loss of linearity.

Limits of detection (LOD) and limits of quantification (LOQ) were calculated as the concentration equivalent to three and 10 times the baseline noise of basal media chromatograms, respectively (n = 48). LOD values for catechin, epicatechin, procyanidin B1, procyanidin B2 and caffeine were 0.019, 0.030, 0.028, 0.030, and 0.043 nmol/mL, respectively (Table 2). These values are 80–360 times lower than a previous HPLC-DAD method for guaraná catechins with detection at 280 nm [2]. The sensitivity of the present method is also a good compromise taking into account the simplicity of the HPLC-UV equipment and its low cost. Our previous method for catechin analysis with a far more expensive coulometric detection [1] is barely 3–6 times more sensitive than the present method, with a basic HPLC-UV setup.

**Table 2:** Limits of detection and quantification for catechins, procyanidins and caffeine in basal media from permeability studies and in vitro digesta of guaraná seed powder, analysed by isocratic HPLC-UV at 210 nm.

Compound	basal media^a^		in vitro digesta^b^	
	LOD (nmol/mL)	LOQ (nmol/mL)	LOD (nmol/mL)	LOQ (nmol/mL)
Catechin	0.019	0.062	0.044	0.148
Epicatechin	0.030	0.100	0.063	0.211
Procyanidin B1	0.028	0.092	0.067	0.223
Procyanidin B2	0.030	0.100	0.073	0.243
Caffeine	0.043	0.144	0.105	0.350

a For injection volumes of 40 μL.

b For injection volumes of 20 μL.

Method accuracy (recovery) was measured after spiking blank in vitro digesta (i.e. the soluble fraction of a blank in vitro digesta) and basal medium of permeation assays (Hanks’ balanced salt solution, HBSS) with 1.56, 12.5 and 50 nmol/mL of catechin, epicatechin and caffeine, and 0.78, 6.25 and 25 nmol/mL of procyanidin B1 and B2, in triplicate. Intraday and interday variation was calculated as relative standard deviation (%RSD) of spiked HBSS and diluted blank in vitro digesta (n = 9), freshly prepared and analysed in two different days by the same analyst. As shown in Table 3, the recovery percentages ranged between 97.2–109.4% for both matrices at all three concentrations, indicating no interactions of the spiked compounds with the components of basal media and in vitro digesta. Repeatability and reproducibility, calculated as intraday and interday %RSD, were all <4% (Table 4), thus within the 10% RSD values acceptable by the ICH guidelines [6].

**Table 3:** Accuracy of the analysis of catechins, procyanidins and caffeine in spiked basal media (HBSS) and in vitro digesta at three concentrations, by isocratic HPLC-UV.

Compound	basal media (recovery %)			in vitro digesta (recovery %)		
	1.56 nmol/mL	12.5 nmol/mL	50 nmol/mL	1.56 nmol/mL	12.5 nmol/mL	50 nmol/mL
Catechin	108.7 ± 0.4	108.8 ± 0.2	109.1 ± 1.0	101.4 ± 0.6	101.0 ± 0.3	101.9 ± 0.6
Epicatechin	108.6 ± 2.1	107.4 ± 0.2	109.4 ± 1.8	100.4 ± 1.4	99.8 ± 0.4	100.7 ± 1.2
Caffeine	104.2 ± 4.6	106.5 ± 0.6	102.5 ± 3.9	98.6 ± 2.8	100.2 ± 0.4	97.2 ± 3.0

	0.78 nmol/mL	6.25 nmol/mL	25 nmol/mL	0.78 nmol/mL	6.25 nmol/mL	25 nmol/mL
Procyanidin B1	107.0 ± 2.1	105.7 ± 0.4	107.6 ± 1.9	103.4 ± 2.4	101.9 ± 0.4	104.6 ± 2.1
Procyanidin B2	103.9 ± 0.5	104.0 ± 0.3	104.1 ± 0.8	98.3 ± 0.9	98.7 ± 0.4	99.1 ± 1.7

**Table 4:** Repeatability (intraday RSD) and reproducibility (interday RSD) of the analysis of catechins, procyanidins and caffeine added to basal media (HBSS) and in vitro digesta, by isocratic HPLC-UV.

Compound	basal media (HBSS)		in vitro digesta	
	Intraday RSD (%)	Interday RSD (%)	Intraday RSD (%)	Interday RSD (%)
Catechin	0.5	2.9	0.5	2.7
Epicatechin	1.3	2.1	1.0	2.3
Caffeine	2.9	1.8	2.1	3.1
Procyanidin B1	1.3	3.8	1.5	2.5
Procyanidin B2	0.5	2.0	1.0	1.1

## Additional information

The present method with detection at 210 nm cannot be used when samples are dissolved in DMSO, methanol, dimethyl formamide or any other solvent with high absorption at low wavelengths. We tried analyzing Caco-2 cell extracts containing methanol, dimethyl formamide and DMSO and they all produce tailing solvent peaks that hindered the detection of catechins at 210 nm. However, only dimethyl formamide efficiently recovered catechins and procyanidins from Caco-2 homogenates. Alternatively, cell homogenates can be extracted and measured at 280 nm, however with a lower sensitivity compared to measurements at 210 nm

## References

[1] Yonekura L., Martins C.A., Sampaio G.R., Monteiro M.P., César L.A.M., Mioto B.M., Mori C.S., Mendes T.M.N., Ribeiro M.L., Arçari D.P., da S. Torres E.A.F. (2016). Bioavailability of catechins from guaraná (Paullinia cupana) and its effect on antioxidant enzymes and other oxidative stress markers in healthy human subjects. Food Funct..

[2] Silva C.P., Sampaio G.R., Freitas R.A.M.S., Torres E.A.F.S. (2018). Polyphenols from guaraná after in vitro digestion: evaluation of bioacessibility and inhibition of activity of carbohydrate-hydrolyzing enzymes. Food Chem..

[3] Monagas M., Padmanabharaju P., Okunji C., Satish J., Reichb E., Giancaspro G. (2018). Development of USP guarana seed monograph family. 18th Annu. Oxford Int. Conf. Sci. Bot..

[4] Machado K.N., de Freitas A.A., Cunha L.H., Faraco A.A.G., de Pádua R.M., Braga F.C., Vianna-Soares C.D., Castilho R.O. (2018). A rapid simultaneous determination of methylxanthines and proanthocyanidins in Brazilian guaraná (Paullinia cupana Kunth.). Food Chem..

[5] Mendes T.M.N., Murayama Y., Yamaguchi N., Sampaio G.R., Fontes L.C.B., da S. Torres E.A.F., Tamura H., Yonekura L. (2019). Guaraná (Paullinia cupana) catechins and procyanidins: gastrointestinal/colonic bioaccessibility, Caco-2 cell permeability and the impact of macronutrients. J. Funct. Foods.

[6] (2005). International Conference on Harmonization of Technical Requirements for Registration of Pharmaceuticals for Human Use. Validation of Analytical Procedures: Text and Methodology, Q2 (R1).

